# The Social Brain: Transcriptome Assembly and Characterization of the Hippocampus from a Social Subterranean Rodent, the Colonial Tuco-Tuco (*Ctenomys sociabilis*)

**DOI:** 10.1371/journal.pone.0045524

**Published:** 2012-09-25

**Authors:** Matthew D. MacManes, Eileen A. Lacey

**Affiliations:** 1 California Institute for Quantitative Biosciences, University of California, Berkeley, California, United States of America; 2 Museum of Vertebrate Zoology and Department of Integrative Biology, University of California, Berkeley, California, United States of America; Cajal Institute, Consejo Superior de Investigaciones Científicas, Spain

## Abstract

Elucidating the genetic mechanisms that underlie complex adaptive phenotypes is a central problem in evolutionary biology. For behavioral biologists, the ability to link variation in gene expression to the occurrence of specific behavioral traits has long been a largely unobtainable goal. Social interactions with conspecifics represent a fundamental component of the behavior of most animal species. Although several studies of mammals have attempted to uncover the genetic bases for social relationships using a candidate gene approach, none have attempted more comprehensive, transcriptome-based analyses using high throughout sequencing. As a first step toward improved understanding of the genetic underpinnings of mammalian sociality, we generated a reference transcriptome for the colonial tuco-tuco (*Ctenomys sociabilis*), a social species of subterranean rodent that is endemic to southwestern Argentina. Specifically, we analyzed over 500 million Illumina sequencing reads derived from the hippocampi of 10 colonial tuco-tucos housed in captivity under a variety of social conditions. The resulting reference transcriptome provides a critical tool for future studies aimed at exploring relationships between social environment and gene expression in this non-model species of social mammal.

## Introduction

Understanding the genetic bases for complex adaptive phenotypes is a fundamental problem in evolutionary biology. In particular, understanding how patterns of gene expression lead to observable differences in phenotypic traits remains a substantial challenge for studies of most organisms. For behavioral biologists, the ability to link variation in gene expression to the occurrence of specific behavioral traits promises to create significant new opportunities to explore the proximate and ultimate bases for variation in animal behavior [1].

Genetic bases for social behavior. - Social interactions represent a fundamental component of the behavior of most animal species [2]–[7]. Given the critical yet complex role that genes play in shaping phenotypic variation, efforts to understand the genetic underpinnings of social interactions are of considerable general interest to behavioral biologists. Studies of gene expression provide a particularly powerful means of linking patterns of behavioral and genetic variation. By comparing rates of gene transcription in individuals that differ with respect to specific phenotypic traits, such studies serve to delineate the genes and gene pathways that underlie the production of particular behavioral phenotypes [8], [9]. Relationships between behavioral variation and patterns of gene expression have been reported for multiple species of insects [10], [11] and birds [9], [12], [13]. Among mammals, such analyses have been used to link aggressive tendencies in mice to differential expression of G-protein coupled neuropeptide receptors such as GABA [14] as well as the loci coding for the proteins septin [15] and calcineurin [16]. Collectively, these studies underscore the potential for analyses of gene expression to elucidate the genetic underpinnings for animal social interactions.

Strategies for studying gene expression. - One approach that has been used to examine the genetic underpinnings for variation in social interactions is to target specific loci (i.e., candidate genes) that are thought to be associated with given behavioral traits [17]. This strategy has been used to explore differences in gene expression in relation to multiple aspects of behavior, including the formation of social bonds [18]–[20]. The primary limitation of this approach is that it requires an *a priori* hypothesis as to which genes are likely to be associated with a given behavioral phenotype information that is lacking for many species and aspects of animal behavior.

An alternative approach makes use of microarrays to identify loci that are differentially expressed in individuals displaying distinct behavioral phenotypes [21]. Microarrays have been employed to examine behaviorally relevant differences in gene expression in model systems like *Drosophila*, thereby uncovering patterns of expression related to mating behavior in post-copulatory females [22], [23] and reproductive success in males [24]. Like the candidate gene approach, however, constructing microarrays requires *a priori* knowledge of the sequences of either the specific gene under investigation [25] or a closely related homolog, either of which limits the suitability of this approach for studies of most organisms.

Transcriptome sequencing and behavior. - The development of high throughput sequencing technologies provides a potential resolution to the challenges posed by the candidate gene and microarray approaches [26], [27]. For studies of complex behavioral traits in particular those thought to be mediated by multiple loci or those for which the underlying genetic bases are unknown high-throughput sequencing of mRNA represents an emerging opportunity to generate either complete or tissue-specific transcriptomes [28], [29] that can be used to quantify differences in gene expression [25]. Importantly, this approach does not require *a priori* knowledge of the genes associated with a given behavioral trait, therefore making it particularly promising for studies of non-model organisms. At the same time, the sensitivity of this approach to even modest differences in gene expression suggests that massively parallel transcriptome sequencing can be used to detect epistatic interactions of the type expected to underlie many complex behavioral phenotypes.

Study system and research objectives. - Given these apparent advantages, we are using transcriptome sequencing to explore the relationship between gene expression and social behavior in the colonial tuco-tuco (*Ctenomys sociabilis*), a group-living subterranean rodent from southwestern Argentina. This species has been the subject of more than 20 years of intensive field research aimed at characterizing the behavior, ecology, and demography of these animals [30], [31]. These studies have revealed that within a single population, *C. sociabilis* displays marked variation in social behavior; while females that disperse as juveniles breed alone as yearlings, females that remain in their natal group rear their young communally during their yearling season [33]. This pronounced intraspecific difference in female behavior provides an important opportunity to investigate patterns of gene expression associated with variation in social relationships minus the effects of potentially confounding differences in ecology and evolutionary history.

Here, we present the results of the first step of this research program a characterization of the transcriptome for *C. sociabilis*. Data were obtained by sequencing RNA from the hippocampi of 10 unrelated yearling females of this species. The hippocampus was chosen for analysis because ongoing studies of the neuroendocrine correlates of social behavior in tuco-tucos have focused on this brain region [32]. In addition to allowing analyses of socially-mediated differences in gene expression, transcriptome sequences will be used to identify SNPs for *C. sociabilis*; because this species currently displays very limited genetic variation [36], SNPs provide the most promising means of assessing the reproductive consequences of behavioral differences among female colonial tuco-tucos. Thus, the transcriptome analyses reported here have multiple important applications to understanding the causes and consequences of variation in social behavior in natural populations of mammals.

## Materials and Methods

### Ethics Statement

All procedures were approved by the University of California, Berkeley, Animal Care and Use Committee and conformed to the guidelines of the American Society of Mammalogists [33] for the use of captive mammals in research.

### Sampling Design

Whole brains were collected from 10 members of a captive population of colonial tuco-tucos (*Ctenomys sociabilis*) housed on the Berkeley campus. This captive population was founded from 12 free-living individuals captured in Neuquen Province, Argentina, in January, 1996. In captivity, the animals were housed in artificial burrow systems constructed of clear Plexiglas tubes connecting several Plexiglas boxes that served as nest chambers and latrines [34]. Typically, the captive population consisted of approximately 45 individuals. Although the social structure and demographic history of *C. sociabilis* [35] have resulted in relatively high levels of inbreeding within natural populations of this species, reproductive partners in the captive population were assigned so as to minimize inbreeding within the study subjects.

Ten unrelated (i.e., non-littermate) yearling females were used in this study. Analyses focused on yearlings because this is the adult age class that is most abundant in nature and that displays the greatest variation in social relationships [36]. Captive females used in this study were housed with 0–3 other adult females, thereby imitating naturally occurring intra-population variation in group size for this species [36]. The social groups in which the test animals were housed were stable for at least one month prior to the collection of brain samples.

Animals were euthanized via overdose with Isoflurane followed immediately by decapitation. The brain was extracted from each individual, placed in a cryotube containing RNAlater (Ambion, Inc), and then flash frozen in liquid nitrogen. Time between death and freezing of tissue did not exceed five minutes.

### RNA Extraction and Sequencing Library Prep

For RNA extraction, brain samples were allowed to thaw in RNAlater in a dedicated, RNAse-free workspace. Once samples were thawed, the hippocampus was dissected out of each brain and placed in fresh RNAlater. Total RNA was extracted using a TRIzol extraction (Invitrogen). Because preparation of an RNA library suitable for sequencing is dependent on having high quality, intact RNA, a small aliquot of each total RNA extract was analyzed on a Bioanalyzer 2100 (Agilent, Palo Alto, CA, USA). RNA integrity was quantified using the RNA Integrity Number (RIN) metric, which was calculated during Bioanalyzer analysis. Following confirmation of sample quality, sequencing libraries were made from each sample using the TruSeq RNA prep kit (Illumina) following the manufacturers instructions. A unique index was ligated to each sample to allow for multiplexed sequencing. The final library was again run on the Bioanalyzer for analysis of fragment size distribution. Each sample was subjected to qPCR using a KAPA kit (Kapa Biosystems, Woburn, MA) for precise quantification. Libraries were then pooled to contain equimolar quantities of each individual library and submitted for Illumina sequencing on a HiSeq 2000 sequencer at The Vincent Coates Genome Sequencing Lab at UC Berkeley. The computer programs used for *de novo* assembly of genome and transcriptome sequences function most efficiently with >20X coverage (i.e., >20 copies of the transcriptome) [37]. Given this recommendation and using the size of the *Mus* transcriptome as a guide, we opted to perform 2 lanes of 150 bp paired-end sequencing.

Assessing sequence quality and pre-assembly procedures. - Accurate assembly of complete transcriptome sequences requires that sequence reads be as error free as possible; random sequencing errors substantially increase the complexity of the de bruijn graph, which may result in assembly error [38], [39]. One potentially important source of error is the failure to delete sequences corresponding to the adapters ligated on to mRNA during library preparation and thus adapter sequences were trimmed (i.e., removed) prior to transcriptome assembly. A second potentially important source of error is a loss of sequence fidelity that frequently occurs near the 3′ end of an Illumina sequencing run. For our analyses, we used a Phred score [40] >20 (corresponding to a 1% sequencing error rate) as our quality threshold and we trimmed all nucleotides falling above this value prior to transcriptome assembly. A third source of error in mRNA sequencing is the non-random incorporation of nucleotides during cDNA priming with hexameric oligonucleotides, as occurs during the initial phases of sequence library construction.

We used the open-source software package Trimmomatic (http://www.usadellab.org/cms/index.php?page=trimmomatic) to identify and to trim nucleotides falling below the established quality threshold as well as to trim adapter sequences. Once sequences were trimmed, we removed reads that were likely to have resulted from routine bacterial contamination of the lab environment (e.g., *E. coli*, NCBI strain 536). To accomplish this, we used the program Bowtie [41] to compare our reads to the genomes of common bacterial contaminants and to remove reads that appeared to be of bacterial origin.

After sequence trimming, we characterized the size (in base pairs) of the DNA molecule subjected to Illumina sequencing, a measure that is required for accurate assembly. Lastly, the GC content of sequencing reads as well as the relationship between GC content and read quality were calculated. Insert size and metrics related to GC content were calculated in the program Picard, available from http://picard.sourceforge.net.

*De novo* sequence assembly was completed using the program Trinity [42], which was run on the Pittsburg Supercomputing Center hardware resource Blacklight (an SGI UV 1000; http://www.psc.edu/machines/sgi/uv/blacklight.php). The raw assembly was filtered using multiple methods. First, transcript quantitation was accomplished following a re-mapping strategy implemented in the program eXpress (http://bio.math.berkeley.edu/eXpress/); this procedure employs read mapping produced using the very-sensitive-local and report all alignments setting in Bowtie 2 [43]. Low confidence contigs were defined as having FPKM values less than 1 and were removed from the dataset. Second, after deleting low confidence contigs, we attempted to remove contig redundancy. This redundancy can be a product of sequencing error, polymorphism, or alternative splicing; because differential splicing may have important phenotypic consequences, only very conservative removal of redundant contigs was attempted. To accomplish this, we used the program cd-hit-est [44], allowing clustering only when 98% sequence similarity occurred. Finally, we searched the database for ribosomal RNA and mitochondrial DNA contamination using a BLAST search against a custom database containing contaminate sequences. Specifically, we made a custom blast database from the contigs remaining after the previous filtration steps. We then downloaded sequence data corresponding to *Cavia* mtDNA and rRNA from Genbank and used a blastn search strategy to identify sequence homology. Any sequences that matched with e <10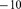 were removed from the dataset.

### Contig Annotation

After *de novo* transcriptome assembly, concatenation, and removal of contaminant reads and duplicate contigs, we subjected the resulting 76,453 contigs (lengths from 300–34,000 bp) to a BLAST search [45] using the NCBI BLAST+ toolkit [45], and a database containing protein sequences from 40 vertebrate species whose genomes have been sequenced (http://uswest.ensembl.org/info/data/ftp/index.html). Matches were considered significant if the e-value for the sequences compared was <10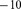. Within the group of significant hits, we chose the best BLAST hits based on percent sequence similarity. In addition, we performed an identical search using the nr database. The results of this search were used to assign GO terms using the Blast2GO software package [46]. GO terms were then clustered into 3 groups: biological process, molecular function, and cellular component. Lastly, using identical search parameters, we used a blast search to compare our dataset against the entire *Cavia* genome.

To assess the potential for contamination by human genomic DNA or RNA in the sequence libraries, we mapped sequence reads to the human genome using Bowtie2, using default parameters. While we expect some small fraction of reads to map due to sequence conservation coupled with the short sequencing read length, the general failure of reads to mapping was interpreted as evidence against human DNA and RNA contamination.

Single nucleotide polymorphisms were identified in the 76k contigs remaining after all sequence editing was complete. Reads from each of the 10 individuals included in this study were mapped to the reference transcriptome (annotated contigs) with the program Bowtie2, using settings identical to the conditions reported above, with the exception of the minimum mapping score, which was increased to 200, and the number of reported alignments, which was reduced to 1. The mpileup [47] program, which is part of the SAMTools [48] package, was used identify SNPs. Putative polymorphisms were retained only if coverage was >40X, the Phred score for each alternative base was >20, and no other polymorphisms were detected within a distance of 300 bp.

## Results

Extraction of total RNA from the hippocampi of the study animals resulted in ∼10 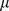
g RNA per individual, of which 4 g was used for Illumina sequencing library prep. Following library construction, ∼700 ng of amplified cDNA per individual remained for use in transcriptome sequencing.

**Figure 1:**
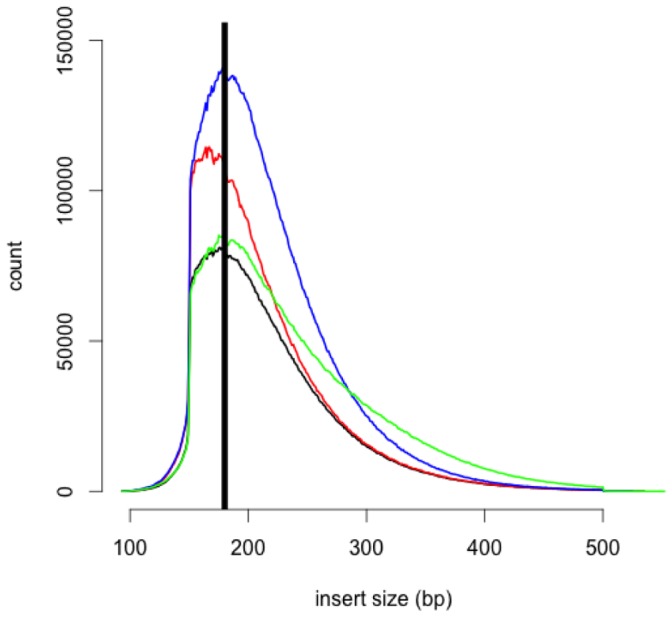
Distribution of insert sizes for libraries sequenced from four *C. sociabilis*. Each individual is represented by a unique color. The vertical black line indicates the most common (modal) insert size.

**Figure 2:**
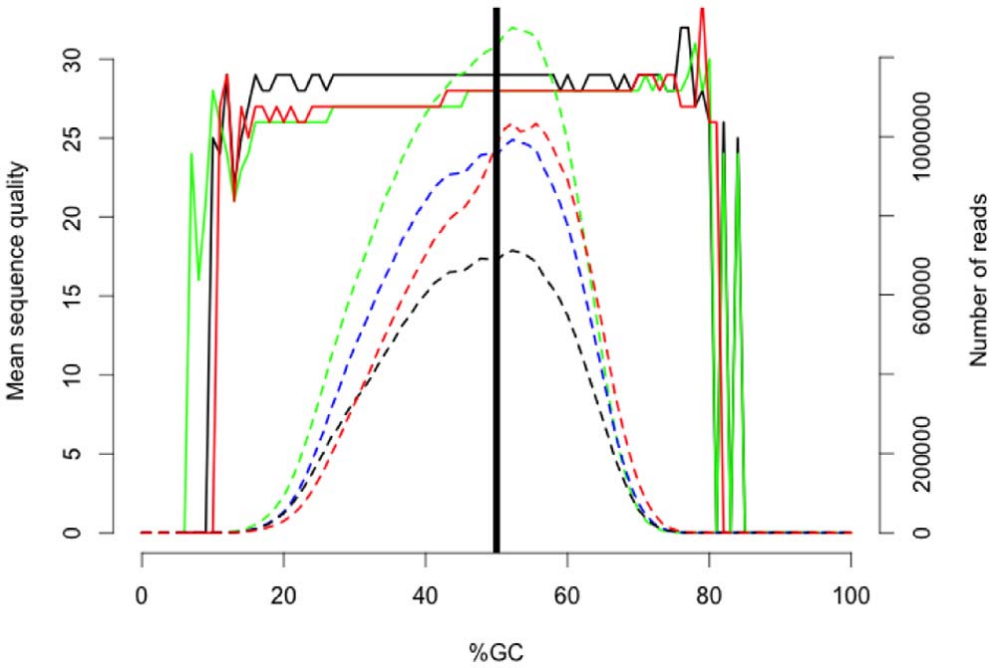
The relationship between %GC content and mean sequence quality (solid lines), as well as the distribution of mean %GC content for the entire 150 bp sequencing read (dashed lines). Each color represents a distinct randomly selected individual library. The colors are consistent between this figure and Figure 1.

Illumina sequencing of 2 lanes required 9 days and resulted in ∼561 million sequencing reads of 150 bp each, which represents a total of ∼200 gigabytes (GB) of raw sequence data; these data are available from the Short Read Archive under accession number SRA051543. Quality trimming resulted in a loss of ∼18% of the total sequence data generated. Comparing our sequence reads to established *E. coli* sequences suggested that ∼0.1% of the remaining reads were bacterial in origin and thus these sequences were also removed from the data set. After all sequence editing was complete, the remaining data set consisted of 476 million sequence fragments for assembly. The mean fragment size was 217 bp, with a mode of 175 bp (Fig. 1). GC content was approximately 50%, with very extreme biases in GC content being most common in low quality sequencing reads (Fig. 2).

**Figure 3:**
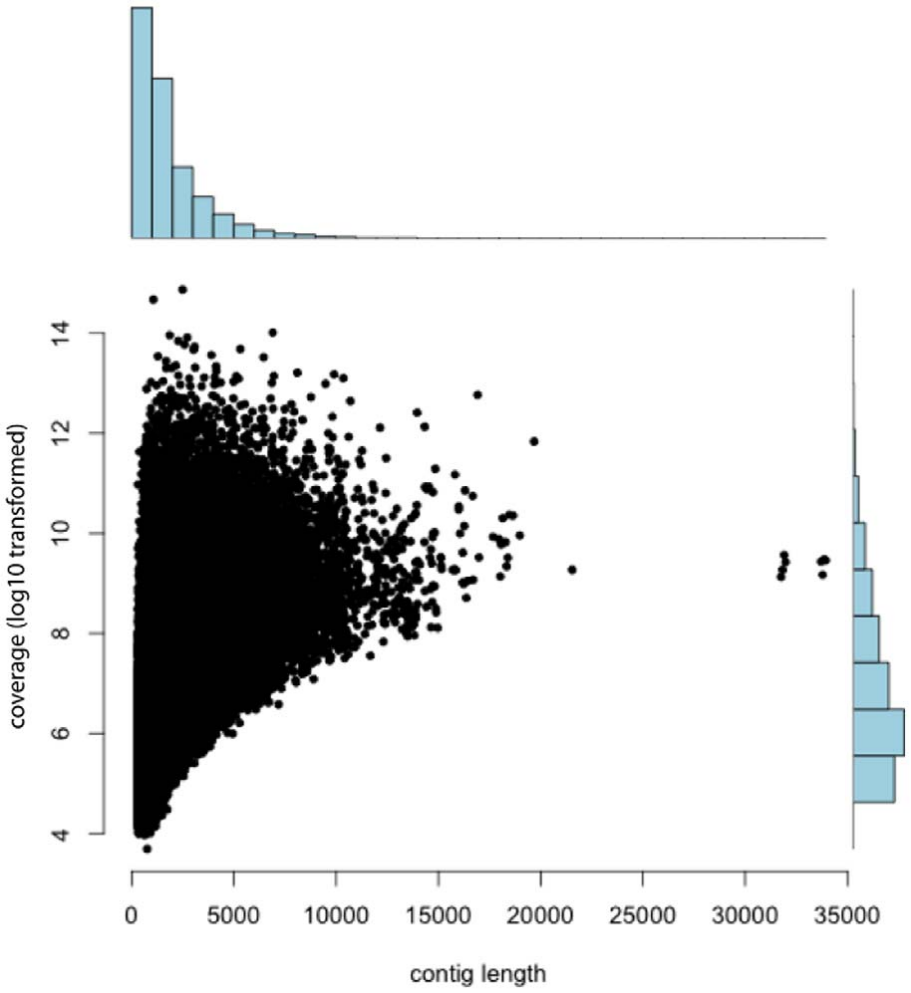
Relationship between sequencing read coverage (log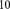 transformed) and contig length, as well as histograms showing the distributions for each axis individually.

Transcriptome assemblies completed in Trinity (kmer length  = 25) were accomplished using approximately 80 cores, 640 Gb RAM, and 336 hours of processing time. The raw assembly was over 700 Mb in size and consisted of 504 k contigs with lengths between 300 bp and 34,000 bp. This dataset was reduced via filtration and the removal of redundancy to 76,453 contigs (300–34,000 bp in length), which sum to approximately 100 Mb of sequence data (Genbank numbers JU497436–JU573888). N50 was 2843 bp, with 15,000 contigs of at least this size (Fig. 3). There was no relationship between contig size and depth of coverage (Fig. 3), indicating that our assembly was probably not limited by depth of sequencing. Over 1,000 high-quality putative SNPs were identified.

**Figure 4:**
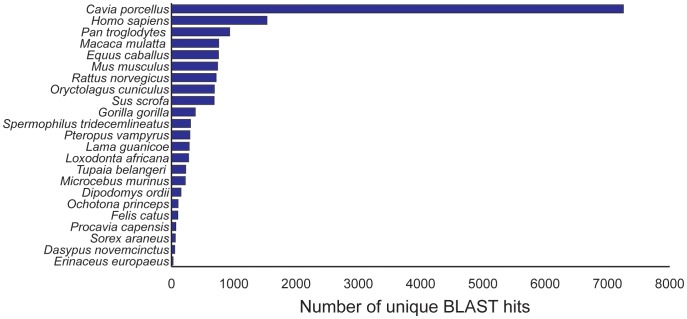
The number of blast hits from 23 mammalian species for which genomes have been sequenced.

Subjecting contigs to BLAST searches (nr, mammal dataset, *Cavia* genome) resulted in the annotation of 50,138 contigs, which represented 66% of the assembled transcriptome. 30,026 contigs matched targets within the mammal databases, with 18,553 of these contigs matching a unique (known) transcript. Gene ontology terms were assigned to contigs, after which contigs were grouped according to their putative relationship to biological processes (table S1), cellular components (table S2), or molecular function (table S3),

Of the ∼18 k contigs that were uniquely annotated to mammalian genes, ∼38% (n = 7256 contigs) best matched known proteins in *Cavia porcellus* (lowest e-value). The remaining contigs matched to a variety of mammalian genera including *Homo* (human), *Mus* (mice), *Rattus* (rats) and *Spermophilus* (ground squirrels) (Fig. 4). For the contigs that matched most closely to non-rodent species, a more restrictive blast search using only *Mus*, *Rattus*, and *Cavia* protein databases uncovered significant matches with 99% of these sequences.

Mapping sequence reads to the Homo genome resulted in a success rate of approximately 5%, which we interpret as strong evidence against contamination. Instead the observation that some contigs BLAST to non-rodent genera (including *Homo*) may be interpreted as a function of the lack of stringency associated with this computational method, rather than evidence of a sample contamination.

Among the annotated transcripts in our data set were multiple loci known to be involved in the regulation of social behavior. These included genes associated with behaviorally important substances such as glucocorticoids [34], GABA [14], [49], [50], septin [15], calcineurin [51], as well as the genes CNTNAP2 (implicated in language delay in autistic children [52]) and FOXP2 (known to be involved in vertebrate communication [17], [53]–[55]). FPKM values for genes in the five most relevant Gene Ontology terms (social behavior, learning or memory, exploration, grooming, and signal transduction) are presented in Table 1.

**Table 1:** Gene Expression.

GO Term	Gene	FPKM	95% CI
exploration behavior	Atxn3	3.081	(2.943107–3.218167)
	Jph3	100.249	(99.712353–100.7861)
	Lrrk2	2.958	(2.893815–3.022012)
	Prkce	31.869	(31.607186–32.131117)
	Tnr	6.997	(6.896392–7.098219)
grooming behavior	Drd2	1.708	(1.623795–1.792786)
	Hprt	10.29	(10.039925–10.540739)
learning or memory	Bdnf	3.589	(3.409089–3.768651)
	Foxp2	5.531	(5.366824–5.696109)
receptors	5HT2c	22.852	(22.544592–23.160105)
	AchR	2.233	(2.009081–2.456789)
	Crhr1	1.035	(0.956885–1.112305)
	Crhr2	1.936	(1.750249–2.121052)
	Gper	1.258	(1.186685–1.328633)
	GR	9.859	(9.649374–10.069106)
	Htr1a	6.175	(6.048717–6.300747)
signal transduction	Cntnap4	4.891	(4.768913–5.013669)
	pelle	1.605	(1.526185–1.684155)
social behavior	Chrnb2	8.326	(8.215878–8.43614)
	Cln8	18.732	(18.43906–19.0245)
	Dlg4	110.376	(109.850226–110.902267)
	Dvl1	5.042	(4.869891–5.213948)
	Gnb1l	2.872	(2.740505–3.003795)
	Grin1	49.725	(49.326893–50.122732)
	Htt	6.564	(6.444615–6.683738)
	Il1b	3.065	(2.901273–3.229691)
	Mapk8ip2	205.936	(202.4805–209.39175)
	Mecp2	8.556	(8.309849–8.801408)
	Nlgn2	39.917	(39.617963–40.215173)
	Nlgn3	6.746	(6.569579–6.921575)
	Npas3	10.778	(10.555809–11.00024)
	Nr2e1	10.438	(9.941466–10.934136)
	Nrxn1	12.449	(12.25531–12.64294)
	Pten	39.738	(39.35365–40.123296)
	Shank1	40.45	(40.112922–40.787768)
	Shank3	31.852	(31.278954–32.425101)
	Tbx1	1.695	(1.492733–1.89637)
	Vps13a	2.485	(2.38346–2.58715)

Gene expression for several genes associated with social behavior, grouped by GO term.

## Discussion

The sequencing strategy employed here was successful in generating a reference transcriptome suitable for use in future studies of gene expression in *C. sociabilis* and other ctenomyid rodents. This sequencing strategy was also successful in identifying >1000 SNPs, thereby providing a valuable toolkit for studies of kinship, reproductive success, and population genetic structure in this species. The transcriptome sequencing strategy described here can be applied to any organism and, unlike candidate-gene or array-based technologies, does not require *a priori* knowledge of the target genome. At the same time, because transcriptome sequencing encompasses all expressed loci, it provides a relatively quick and efficient means of identifying multiple genes that may be associated with a given pattern of phenotypic variation.

Previous studies of *C. sociabilis* have revealed that this species is characterized by remarkably little genetic variation throughout its geographic range [35], [56], [57]. As a result, efforts to characterize the reproductive consequences of differences in social behavior have proven largely unsuccessful [31]. For the same reason, efforts to explore the population genetic structure of these animals have been limited (but see [35]). The latter constraint is particularly relevant given the recent (2012) eruption of the Puyehue-Cordon Caulle volcanic chain, ash fall from which blanketed the entire geographic range of *C. sociabilis*; coupled with the long-term demographic data available for this species [36], this eruption provides a rare opportunity to document directly the effects of a catastrophic natural event on the genetic structure of natural populations of mammals. Development of the SNP markers identified during this study provides a probably means of quantifying genetic variation in *C. sociabilis* over small spatial and temporal scales, thereby potentially allowing investigation of these and other critical behavioral and demographic themes.

Future studies of *C. sociabilis* and other ctenomyid rodents will use the reference transcriptome described here to examine differences in gene expression between solitary and group-living conspecifics. In contrast to other studies of the genetics of social behavior, most of which employ comparisons made at higher taxonomic levels (i.e. [58]), the planned analyses of *C. sociabilis* will capitalize upon naturally occurring intraspecific variation in social environment. In the free-living population of this species that has been the subject of long-term field research, both lone and group-living yearling females are present during each annual breeding season [30], [36], [59]. This natural variation in the social environments experienced by conspecifics provides an ideal opportunity to use transcriptome sequencing to identify differences in gene expression associated with adaptively significant differences in social environment. Such intraspecific comparisons are particularly valuable because they are not subject to the potentially confounding variables (e.g., differences in ecology, phylogenetic history) that affect interspecific analyses. As a result, transcriptome-based analyses of gene expression in *C. sociabilis* should prove highly informative regarding the genetic bases for variation in social behavior in this species.

## Supporting Information

Table S1 A list of all GO terms grouped by biological process. Column 1 is the GO ID, followed by the GO term description, followed by the number of sequences assigned to this term in column 3.
(TXT)

Table S2 A list of all GO terms grouped by cellular component. Column 1 is the GO ID, followed by the GO term description, followed by the number of sequences assigned to this term in column 3.
(TXT)

Table S3 A list of all GO terms grouped by molecular function. Column 1 is the GO ID, followed by the GO term description, followed by the number of sequences assigned to this term in column 3.
(TXT)
